# First born children are taller, insulin resistant and have higher blood pressure

**DOI:** 10.1186/1687-9856-2013-S1-O53

**Published:** 2013-10-03

**Authors:** Tim Savage, Ahila Ayyavoo, Paul Hofman, Jose Derraik, Wayne Cutfield

**Affiliations:** 1 The Liggins Institute, University of Auckland, New Zealand

## Background

There is a strong trend towards couples having fewer children, leading to a marked increase in the population of first born compared to later born children.

## Aims and hypotheses

We aimed to determine if birth order influences childhood physical or metabolic parameters. We hypothesised that first born children would have a different auxological and metabolic profile to later born children.

## Methods

Prepubertal children aged 3–10 years, (born at full term and not SGA) were recruited and grouped by birth order. All children had height, weight, body composition by DEXA scan and fasting serum IGF-1, IGF-2, IGFBP3, lipids, leptin, adiponectin, glucose and insulin levels recorded. Children's heights and BMI were expressed as an SDS, and corrected for mean parental height and BMI SDS. A subgroup of children underwent 24 hr ambulatory BP monitoring and a frequently sampled IVGTT to determine insulin sensitivity with the minimal model. Results are expressed as mean ±SEM.

## Results

410 children (mean age 7.4±0.9 years) were studied: 191 first, 159 second, and 60 third borns. First borns were taller than later borns (HtSDS 0.42±0.06 vs 0.13±0.07 p=<0.0001) when adjusted for their genetic height potential. First borns were taller than second borns (0.43±0.06 vs 0.19±0.07 SDS p=0.002); second borns were taller than third borns (0.19±0.07 vs -0.14±0.11 p=0.01). First borns were also taller than third borns (p< 0.001).

A sub-group (n=90) underwent precise measurement of insulin sensitivity and 24 hour ambulatory BP monitoring comprised of 34 first borns and 56 later borns. First borns were less insulin sensitive than later borns (Si 10.7±0.82 vs 13.10±0.94, p=0.033). 24 hour ambulatory blood pressure monitoring in these children revealed that first borns had higher daytime systolic (113.3±3.1 vs108.8±2.6 mmHg p=0.03) and higher day (68.4±2.1vs 64.7±1.7 mmHg p=0.01) and night-time (55.2±2.3 vs 53.7±1.9mmHg p=0.01) diastolic blood pressures than later borns.

## Conclusions

First born children are taller than later borns. Birth order also has a graded effect on height, with incremental height reduction from first to third birth order. We found that first born children have reduced insulin sensitivity and higher blood pressure. This suggests that being first born is a risk factor for these two major components of the metabolic syndrome.

